# ﻿Three new species and a new record of ConocybesectionPilosellae (Bolbitiaceae, Agaricales) from Jilin Province, China

**DOI:** 10.3897/mycokeys.114.140056

**Published:** 2025-02-24

**Authors:** Han-bing Song, Tolgor Bau

**Affiliations:** 1 Key Laboratory of Edible Fungal Resources and Utilization (North), Ministry of Agriculture and Rural Affairs, Jilin Agricultural University, Changchun 130118, Jilin, China Jilin Agricultural University Changchun China

**Keywords:** *Conocybe* section *Pilosellae*, morphology, new taxa, phylogeny

## Abstract

This study is based on the phylogenetic framework of ConocybesectionPilosellae and incorporates materials from Jilin Province. A systematic phylogenetic tree was constructed using maximum likelihood and Bayesian analyses of internal transcribed spacer region (ITS) and nuclear large subunit ribosomal DNA (nrLSU), and translation elongation factor 1-alpha (*tef1-α*) sequences. As a result, three new species were discovered in Jilin Province: *Conocybeverna*, which emerges in broad-leaved forests during spring; *C.angulispora*, characterized by angular and submitriform or slightly hexagonal basidiospores; and *C.rubrocyanea*, with basidiomata displaying a reddish hue when fresh and a bluish hue when dry. Additionally, a new record for China, *C.hexagonospora* was identified, characterized by the lack of distinct pubescence on the pileus and slightly hexagonal basidiospores, increasing the total number of species within sect. Pilosellae to 22. Key for sect. Pilosellae is provided, accompanied by morphological descriptions and line drawings for the new species and a new record for China.

## ﻿Introduction

*Conocybe* Fayod belongs to the family Bolbitiaceae Singer and was established by Fayod 1889. Its taxonomic status has undergone multiple revisions and clarifications (Fries 1821; Fayod 1889; Kühner 1935; Singer 1949; Watling 1982). Conocybesect.Pilosellae Singer, a basal group of *Conocybe*, was established by Singer in 1962 based on stipes with hairs and non-lecythiform caulocystidia (Singer 1962). The classification history has been summarized by Song and Bau (2023), and we won’t go into further detail here. Please refer to the following references for more information (Kühner 1935; Singer 1949; Singer 1962; Watling 1982; Bon 1992; Arnolds 2005; Hausknecht 2005; Hausknecht and Krisai-Greilhuber 2006). Hausknecht and Krisai-Greilhuber (2006) divided sect. Pilosellae into two subsections, based on the size and shape of basidiospores, the presence of lecythiform caulocystidia, and habitat. The two subsections are subsect. Pilosellae and subsect. SiligineaeHauskn. & Krisai.Subsect.Pilosellae includes seven series: ser. Pilosella, ser. SienophyllaHauskn. & Krisai,ser.Anthracophila Hauskn. & Krisai, ser. BisporaHauskn. & Krisai,ser.Microrrhiza Hauskn. & Krisai, ser. InocybeoidesHauskn. & Krisai, andser.CylindraceaHauskn. & Krisai.SubsectionSiligineae Hauskn. & Krisai includes four series: ser. SiligineaHauskn. & Krisai,ser.Fimetaria Hauskn. & Krisai, ser. MurinaceaHauskn. & Krisai, andser.Lenticulospora Hauskn. & Krisai (Hausknecht and Krisai-Greilhuber 2006; Hausknecht 2009). Tóth et al. (2013) included members of sect. Pilosellae in a molecular phylogenetic analysis of Bolbitiaceae based on combined dataset of ITS, nrLSU, and *tef1-α* sequences, providing important reference data for subsequent phylogenetic studies of Bolbitiaceae. Species of sect. Pilosellae have a wide distribution and are mostly found in fertile soil and herbivore dung (Hausknecht 2009). They contain toxic substances such as psilocybin, phallotoxins, and amatoxins, and have certain pharmacological value (Griffiths et al. 2016; Johnson et al. 2017; Paul 2021).

In China, research on *Conocybe* began with Tai (1979). As of 2023, a total of 40 species of *Conocybe* have been recorded in China, including 17 species in sect. Pilosellae (Tai 1979; Xie et al. 1986; Li et al. 1993; Bi et al. 1994; Yuan and Sun 1995; Zhang and Mao 1995; Li and Bau 2003; Li and Azbukina 2011; Bau et al. 2014; Li et al. 2015; Wang and Tzean 2015; Bau 2016; Liu and Bau 2018; Liu 2018; Zhang 2019; Ye 2021; Song et al. 2023; Song and Bau 2023). However, it is important to note that the majority of these species are distributed in Northeast China.

Jilin Province is characterized by a temperate monsoon climate with distinct seasons. The summers are rainy and warm, while the winters are dry and cold. The region’s main geographical features include mountains and plains. Forested areas are mainly concentrated in the Changbai Mountains, where diverse vegetation types, such as mixed coniferous and broadleaf forests, dominate. The region’s excellent natural environment and abundant vegetation provide favorable conditions for fungal diversity. However, the number of reported species of sect. Pilosellae in Jilin province is significantly lower compared to Europe and North America at the same latitude. This indicates the need for further investigation and research. Based on results from this study, the number of species of sect. Pilosellae species in China is increased to 22 (include *C.siliginea*, collected from Henan Province).

## ﻿Materials and methods

### ﻿Samplings and morphological analyses

The specimens for this study were collected from Jilin Province, China, from 2022 to 2023. Upon discovery, photographs were taken, and information on habitat and morphological features was recorded. Subsequently, the materials were dried using silica gel desiccants, prepared as specimens, and stored at the Fungarium of Jilin Agricultural University (FJAU). To examine the microscopic structures of the specimens, they were treated with a 5% KOH solution and a 1% Congo red solution. Reacting with lamellar structures using a 25% ammonia solution (Hausknecht 2009). The observations were made using a Carl Zeiss Primo Star optical microscope from Jena, Germany. Additionally, the color of fresh or dried basidiomata was described using the color-coding system developed by the German Institute for Quality Assurance and Certification (Reichs-Ausschuss fur Lieferbedingungen und Guetesicherung, available at https://www.ral-guetezeichen.de/), the abbreviation used in the text (RAL).

In this study, the basidiospore measurements do not include the apiculus. They are presented as ‘(a)b–c(d)’, where ‘b–c’ represents the minimum at least 90% of the measured values, and ‘a’ and ‘d’ represent the extreme values. To accurately record their dimensions, the main body (excluding sterigmata or excrescences) of the basidia, cheilocystidia, caulocystidia, and pileipellis were measured if present. At least 20 were measured. The notation (n/m/p) indicates that the measurements were made on “n” randomly selected basidiospores from “m” basidiomata of “p” collections. Twenty basidiospores are measured from each basidioma. This sampling method ensures a representative measurement sample. The ratio of length divided by width, known as Q, provides a measure of the elongation of the spores. The average quotient (length/width and breadth), denoted as Qm, is calculated along with the standard deviation to provide an overall average value with variation.

### ﻿DNA extraction, PCR amplification, and sequencing

To extract total genomic DNA from the dried specimens, we followed the manufacturer’s instructions and used a NuClean Plant Genomic DNA kit (ComWin Biotech, CW0531M, Taizhou, China). For amplifications, we employed the primer pairs ITS1F/ITS4 (White et al. 1990; Gardes and Bruns 1993), LR0R/LR7 (Vilgalys and Hester 1990; Moncalvo et al. 2000), and EF1-983F/EF1-2218R (Rehner and Buckley 2005) for the ITS, nrLSU, and tef1-α sequences, respectively. Polymerase chain reaction (PCR) amplification was conducted on a Bio-Rad T100TM Thermal cycler (Bio-RAD Inc., Hercules, CA, USA). In a 30 µL reaction mixture, we used the following final concentrations or total amounts: 2 µL of template DNA, 15 µL of 2× SanTaq PCR Master Mix (B532061, Sangon Biotech, Shanghai, China), 1.5 µL of each primer, and 10 µL of double-distilled water (ddH_2_O).

The PCR protocol for ITS and nrLSU involved the following conditions: initial denaturation at 94 °C for 5 min, followed by 35 cycles of denaturation at 94 °C for 30 s, annealing at 53 °C (ITS, nrLSU) for 30 s, and extension at 72 °C for 45 s (ITS)/80 s (nrLSU). The final extension was performed at 72 °C for 10 min, followed by cooling at 4 °C indefinitely. For *tef1-α*, the touchdown PCR protocol was as follows: initial denaturation at 94 °C for 3 min, followed by 8 cycles of denaturation at 94 °C for 40 s, annealing at 60 °C for 40 s (with the temperature decreasing by 1 °C per cycle), and extension at 72 °C for 2 min. After the initial cycles, the denaturation step was repeated at 94 °C for 45 s, followed by annealing at 60 °C for 40 s and extension at 72 °C for 2 min, repeated for a total of 36 cycles. Finally, a final extension step was performed at 72 °C for 10 min and cooling at 15 °C indefinitely.

Following the PCR amplification, the products were electrophoresed on a 1% agarose gel along with known standard DNA markers. The resulting PCR products were sent for sequencing services to Sangon Biotech (Shanghai) Co., Ltd., and sequence data was obtained. To ensure the quality of the chromatograms, they were checked in BioEdit v7.2.5 (Hall 1999), ensuring that each base was of good quality. Additionally, a BLAST search was conducted using the National Center of Biotechnology Information (NCBI) database to confirm that the sequencing results matched the specimens. Finally, the sequences were submitted to GenBank.

### ﻿Phylogenetic analyses

Sequences were downloaded from GenBank (Table 1). The ITS, nrLSU, and *tef1-α* sequences were aligned using the G-INS-i algorithm with two iterative cycles only via the online Mafft tool (Katoh et al. 2019; https://mafft.cbrc.jp/alignment/server/). The resulting alignment was then manually refined and trimmed using MEGA7 (Kumar et al. 2016). To generate the concatenated alignment, PhyloSuite 1.2.2 (Zhang et al. 2020) was employed. The best-fit partition model (edge-unlinked) was selected using the BIC criterion with ModelFinder v2.2.0 (Kalyaanamoorthy et al. 2017). For maximum likelihood phylogenies, IQ-TREE was used under the Edge-linked partition model for 1000 standard bootstraps, along with the Shimodaira-Hasegawa-like approximate likelihood-ratio test, with settings based on the results from ModelFinder (Nguyen et al. 2015; Guindon et al. 2010). Bayesian inference phylogenies were inferred using MrBayes 3.2.7a under the partition model (Ronquist et al. 2012) through two parallel runs (MCMC) and 4,500,000 generations, discarding the initial 25% of the sampled data as burn-in, average standard deviation of split frequencies is 0.009. Finally, the figures were edited using iTOL (Letunic and Bork 2019), Adobe Photoshop 2021, and Adobe Illustrator 2021. The outgroup used was the *Psathyrella* species (Song and Bau 2023).

**Table 1.** Information on the DNA sequences used to reconstruct phylogenetic trees. Sequences in bold were newly generated in this study. T = holotype.

Taxon	Voucher specimen	GenBank accession numbers			Origin	References
		ITS	nrLSU	*tef1-α*		
*Bolbitiuscoprophilus*	HMJAU64958	OQ780315	OQ758216	–	China	Song and Bau 2023
*B.coprophilus*	SZMC-NL-2640	JX968253	JX968370	–	Hungary	Tóth et al. 2013
*B.reticulatus*	WU30001	JX968249	JX968366	JX968455	Hungary	Tóth et al. 2013
*B.subvolvatus*	WU28379	JX968248	JX968365	JX968454	Italy	Tóth et al. 2013
*Conocybealkovii*	LE262841	JQ247196	–	–	Russia	Malysheva 2012
***C.angulispora***	**FJAU65120 T**	**PP501383**	**PP501393**	**PP501651**	**China**	**This study**
***C.angulispora***	**FJAU65122**	**PP501384**	**PP501394**	**PP501652**	**China**	**This study**
C.anthracophilavar.ovispora	WU25461	JX968237	JX968355	–	Italy	Tóth et al. 2013
*C.antipus*	WU19791	JX968215	JX968332	JX968432	Austria	Tóth et al. 2013
*C.bispora*	SZMC-NL-2573	JX968203	JX968320	JX968423	Hungary	Tóth et al. 2013
*C.bisporigera*	SZMC-NL-1904	JX968235	JX968353	JX968446	Hungary	Tóth et al. 2013
*C.brachypodii*	HMJAU45017	MH141423	–	–	China	Liu 2018
*C.brunneidisca*	HMJAU45069	OQ780317	–	–	China	Song and Bau 2023
*C.ceracea*	HMJAU64951	OQ758110	OQ758218	OQ758305	China	Song and Bau 2023
*C.coniferarum*	LE313009	KY614061	–	–	Russia	Malysheva 2017
*C.crispella*	WU27367	JX968208	JX968325	JX968426	Australia	Tóth et al. 2013
*C.cylindracea*	WU20796	JX968240	JX968358	JX968449	Italy	Tóth et al. 2013
*C.cylindrospora*	HMJAU42440	MG250375	OQ758203	–	China	Liu and Bau 2018; Song and Bau 2023
*C.deliquescens*	HMJAU61998	OP373403	OQ758204	OQ758292	China	Song and Bau 2023
*C.elegans*	SZMC-NL-0908	JX968223	JX968341	JX968437	Sweden	Tóth et al. 2013
*C.enderlei*	WU21272	JX968163	JX968279	–	Italy	Tóth et al. 2013
*C.fuscimarginata*	HMJAU45033	OQ780310	OQ758208	OQ758296	China	Song and Bau 2023
*C.fuscimarginata*	SZMC-NL-3668	JX968238	JX968356	JX968448	Sweden	Tóth et al. 2013
*C.gigasperma*	SZMC-NL-3972	JX968179	JX968295	JX968403	Slovakia	Tóth et al. 2013
*C.hausknechtii*	LE253789	JQ247194	–	–	Russia	Malysheva 2013
***C.hexagonospora***	**FJAU71661**	**PP501385**	**PP501395**	**PP501653**	**China**	**This study**
*C.hydrophila*	HMJAU64954	OQ758116	OQ758232	OQ758313	China	Song and Bau 2023
*C.incarnata*	FJAU71663	PP501390	PP501400	PP501658	China	Song and Bau 2023
*C.incarnata*	WU21897	JX968229	JX968347	JX968441	Finland	Tóth et al. 2013
*C.incerta*	LE313017	KY614062	–	–	Russia	Malysheva 2017
*C.ingridiae*	WU28158	JX968244	JX968361	JX968451	Italy	Tóth et al. 2013
*C.karakensis*	KTK05	ON392730	–	–	Pakistan	Ullah et al. 2023
*C.lenticulospora*	SZMC-NL-0923	JX968242	JX968359	JX968450	Sweden	Tóth et al. 2013
*C.mesospora*	HMJAU45049	MH141419	–	–	China	Liu 2018
*C.microrrhiza*	SZMC-NL-2180	JX968222	JX968340	JX968436	Hungary	Tóth et al. 2013
*C.moseri*	GLM-F40421	MK412354	–	–	Germany	Unpublished
*C.moseri*	HMJAU45075	OQ780309	OQ758207	–	China	Song and Bau 2023
*C.muscicola*	HMJAU64939	OQ758113	OQ758223	OQ758309	China	Song and Bau 2023
*C.nigrescens*	WU27557	JX968234	JX968352	JX968445	Italy	Tóth et al. 2013
*C.nitrophila*	WANG140019	KR998384	–	–	China	Wang and Tzean 2015
*C.nitrophila*	WU20916	JX968233	JX968351	JX968444	India	Tóth et al. 2013
C.ochrostriatavar.favrei	WU29786	JX968245	JX968362	JX968452	Italy	Tóth et al. 2013
*C.olivaceopileata*	LE313106	KY614059	–	–	Russia	Malysheva 2017
*C.pallidospora*	WU7395	JX968239	JX968357	–	Austria	Tóth et al. 2013
*C.parapilosella*	90551	MN872706	–	–	Spain	Siquier and Salom 2021
*C.pilosella*	HMJAU45062	OQ780305	OQ758205	OQ758294	China	Song and Bau 2023
*C.pilosa*	HMJAU64947	OQ758122	OQ758222	OQ758307	China	Song and Bau 2023
*C.praticola*	HMJAU64965	OQ780303	–	–	China	Song and Bau 2023
*C.pseudocrispa*	HMJAU64946	OQ780307	OQ758212	OQ758293	China	Song and Bau 2023
*C.pseudocrispa*	WU18009	JX968230	JX968348	JX968442	Austria	Tóth et al. 2013
*C.pubescens*	WU20759	JX968170	JX968286	JX968396	Italy	Tóth et al. 2013
*C.reniformis*	HMJAU64942	OQ758108	OQ758229	OQ758311	China	Song and Bau 2023
*C.rickenii*	AH21067	MF142238	–	–	Spain	Siquier and Salom 2018
*C.romagnesii*	HMJAU64960	OQ780304	–	–	China	Song and Bau 2023
*C.rostellata*	SZMC-NL-2499	JX968162	JX968278	JX968390	Sweden	Tóth et al. 2013
*C.rubrocyanea*	HMJAU64964	OQ749742	–	–	China	Song and Bau 2023
***C.rubrocyanea***	**FJAU65123 T**	**PP501388**	**PP501398**	**PP501656**	**China**	**This study**
***C.rubrocyanea***	**FJAU71654**	**PP501389**	**PP501399**	**PP501657**	**China**	**This study**
*C.rufostipes*	HMJAU64937	OQ758120	OQ758227	OQ758317	China	Song and Bau 2023
*C.semiglobata*	WU8794	JX968188	JX968304	–	Austria	Tóth et al. 2013
*C.siennophylla*	HMJAU64966	OQ780312	OQ758210	OQ758297	China	Song and Bau 2023
*C.siennophylla*	SZMC-NL-1210	JX968246	JX968363	JX968453	Hungary	Tóth et al. 2013
*C.siliginea*	SZMC-NL-2313	JX968225	JX968343	JX968438	Sweden	Tóth et al. 2013
***C.siliginea***	**FJAU71664**	**PP501392**	**PP501402**	**PP501660**	**China**	**This study**
*C.singeriana*	HMJAU64956	OQ780314	OQ758214	–	China	Song and Bau 2023
*C.singeriana*	WU22129	JX968166	JX968282	JX968393	Austria	Tóth et al. 2013
*C.sinobispora*	HMJAU64949	OQ758118	OQ758230	OQ758315	China	Song and Bau 2023
*Conocybe* sp.1	HMJAU44988	OQ749737	OQ740305	OQ758302	China	Song and Bau 2023
*Conocybe* sp.2	HMJAU64963	OQ749740	OQ740307	OQ758304	China	Song and Bau 2023
*Conocybe* sp.3	HMJAU64967	OQ749741	–	–	China	Song and Bau 2023
*C.tetrasporoides*	WU17385	JX968232	JX968350	–	New Zealand	Tóth et al. 2013
*C.velutinomarginata*	WU28695	JX968226	JX968344	JX968439	Germany	Tóth et al. 2013
***C.velutipes***	**FJAU71662**	**PP501391**	**PP501401**	**PP501659**	**China**	**This study**
*C.velutipes*	SZMC-NL-2187	JX968228	JX968346	JX968440	Hungary	Tóth et al. 2013
***C.verna***	**FJAU65117 T**	**PP501386**	**PP501396**	**PP501654**	**China**	**This study**
***C.verna***	**FJAU65118**	**PP501387**	**PP501397**	**PP501655**	**China**	**This study**
*C.volvicystidiata*	LIP0001212	KY346827	–	–	France	Hausknecht and Broussal 2016
*C.watlingii*	WU22744	JX968172	JX968288	JX968398	Finland	Tóth et al. 2013
*C.nocybula.coprophila*	SZMC-NL-2176	JX968156	JX968273	–	Hungary	Tóth et al. 2013; Song and Bau 2024
*C..cyanopus*	WU2134	JX968157	JX968274	JX968388	Austria	Tóth et al. 2013; Song and Bau 2024
*C..smithii*	HMJAU62001	OP373407	OQ758215	OQ758300	China	Song and Bau 2023; Song and Bau 2024
*Conobolbitinadasypus*	SZMC-NL-2279	JX968152	JX968269	JX968385	Hungary	Tóth et al. 2013; Song and Bau 2024
*Descoleaantarctica*	NZ5182	AF325647	–	–	USA	Peintner et al. 2001
*D.quercina*	HMJAU64959	OQ780313	OQ758213	OQ758299	China	Song and Bau 2023
*Pholiotinaarrhenii*	SZMC-NL-2509	JX968261	JX968377	–	Sweden	Tóth et al. 2013
*Ph.brunnea*	SZMC-NL-1216	JX968259	JX968375	JX968461	Hungary	Tóth et al. 2013
*Ph.dentatomarginata*	SZMC-NL-2921	JX968258	JX968374	JX968460	Hungary	Tóth et al. 2013
*Ph.serrata*	HMJAU62006	OP538570	OQ758217	OQ758301	China	Song and Bau 2023
*Ph.sulcata*	SZMC-NL-1975	JX968153	JX968270	JX968386	Hungary	Tóth et al. 2013
*Ph.teneroides*	SZMC-NL-3501	JX968264	JX968379	JX968465	Slovakia	Tóth et al. 2013
*Ph.utricystidiata*	WU20164	JX968262	JX968463	–	Germany	Tóth et al. 2013
*Ph.vexans*	SZMC-NL-3967	JX968265	JX968380	JX968466	Slovakia	Tóth et al. 2013
*Psathyrellaleucotephra*	SZMC-NL-1953	FM163226	FM160683	FM897219	Hungary	Nagy et al. 2011
*P.piluliformis*	HMJAU37922	MG734716	MW413364	MW411001	China	Yan and Bau 2018

## ﻿Results

### ﻿Phylogenetic analyses

**Figure 1.**
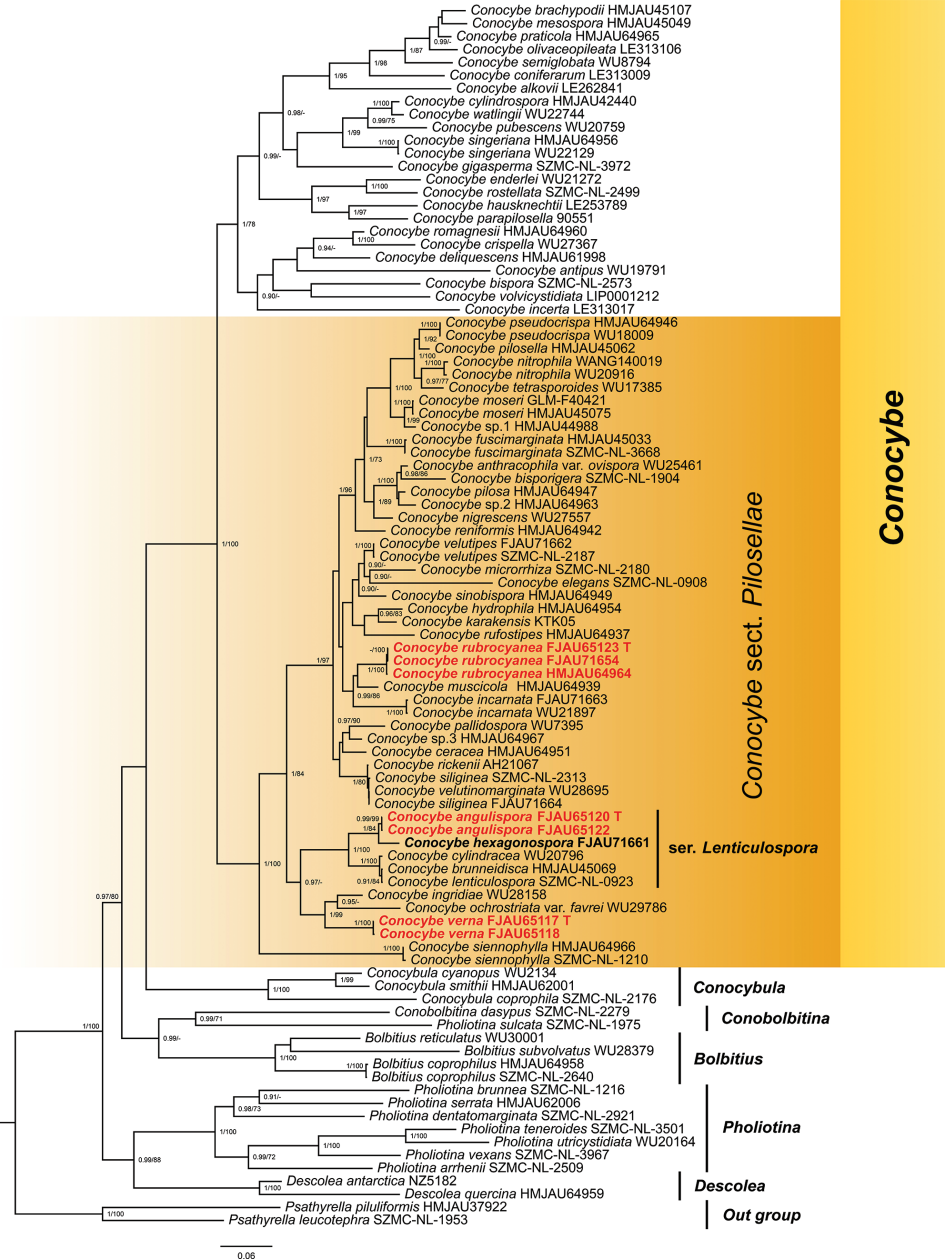
The phylogenetic relationships of Conocybesect.Pilosellae in Bolbitiaceae using Bayesian inference and maximum likelihood methods based on a multi-locus dataset (ITS, nrLSU, and *tef1-α*). In the phylogenetic tree, the newly proposed species are indicated in bold red color, while the newly recorded species is indicated in bold black color, the outgroup is *Psathyrella* species, T = holotype.

The Bayesian tree was constructed based on a combined dataset of ITS, nrLSU, and *tef1-α*, while the ML phylogenetic tree was not presented due to their similar topology. Bootstrap support values were indicated on the tree nodes. Only the data meeting the criteria of Bayesian posterior probabilities (PP ≥ 0.9) and ML bootstrap values (MLbs ≥ 70%) were retained (Fig. 1). The multi-locus dataset (ITS + nrLSU + *tef1-α*) of *Conocybe* comprised 826 bp for ITS, 1299 bp for nrLSU, and 1131 bp for *tef1-α*. The alignment included 94 sequences with 3256 columns, resulting in 1446 distinct patterns, 997 parsimony-informative sites, 316 singleton sites, and 1943 constant sites. During the construction of ML phylogenetic trees, the best-fit models, GTR+F+R4 for ITS, TIM3+F+I+I+R2 for nrLSU, and TIM2e+I+I+R4 for *tef1-α* based on the BIC. Similarly, for Bayesian phylogenetic trees, the best-fit models according to the BIC were GTR+F+I+G4 for ITS and nrLSU, and SYM+I+G4 for *tef1-α*.

In the phylogenetic tree, the newly proposed species are indicated in bold red color, while the newly recorded species is indicated in bold black color (Fig. 1). Notably, specimens FJAU65123, FJAU71654, and HMJAU64964 clustered together, forming a distinct branch and serving as sister taxa to *Conocybemuscicola* T. Bau & H.B. Song. However, their phylogenetic relationship exhibits low support, with a PP/MLbs value of 0.7/55. Similarly, FJAU65120 and FJAU65122 comprise a separate branch and act as sister taxa to *C.hexagonospora* Métrod ex Hauskn. & Enderle (FJAU71661), demonstrating a PP/MLbs value of 1/84. Furthermore, FJAU65117 and FJAU65118 form another distinct branch and serve as sister taxa to *C.ingridiae* Hauskn. and C.ochrostriatavar.favrei Hauskn., with a PP/MLbs value of 1/99. We conducted a Standard Nucleotide BLAST of the ITS sequences of *Conocybeverna* (FJAU65117), *C.angulispora* (FJAU65120), and *C.rubrocyanea* (FJAU65123) against the NCBI database. The results, presented in descending order of similarity, showed that *C.verna* had a similarity of 96.7% with Conocybecf.rostellata (SMNS-STU-F-0900917), 92.8% with *C.ingridiae* (WU28158), and 93.1% with C.ochrostriatavar.favrei (WU29786). *Conocybeangulispora* exhibited a similarity of 98.2% with *C.lenticulospora* (HMJAU45069), 98.6% with *C.cylindracea* (WU20796), and 88.7% with *C.hydrophila*. The similarity between *C.rubrocyanea* and *C.muscicola* (HMJAU64939) was 95.2%, with *C.velutipes* (SZMC-NL-2187) was 94.4%, and with *C.fuscimarginata* (HMJAU45033) was 93.8%. And then based on the phylogenetic tree and morphological findings, three new species are proposed: *C.rubrocyanea* (for the clade FJAU65123, FJAU71654, and HMJAU64964), *C.angulispora* (for the clade FJAU65120 and FJAU65122), and *C.verna* (for the clade FJAU65117 and FJAU65118). While the type specimen of *C.hexagonospora* lacks sequence data, the identification of this species as a new record for China was accomplished through traditional morphology, and reference sequences have been provided to facilitate future confirmation. Finally, the branch containing *C.angulispora*, *C.hexagonospora*, *C.cylindracea* Maire & Kühner ex Singer, and *C.brunneidisca* (Murrill) Hauskn. referred to as ser. Lenticulospora, following the viewpoint of Hausknecht and Krisai-Greilhuber (2006) (for more detailed information, please refer to the Discussion section).

### ﻿Taxonomy

#### ﻿ Conocybe verna

Taxon classificationFungiAgaricalesBolbitiaceae

T. Bau & H. B. Song sp. nov.

F693FEB4-8EDE-5A6B-A93F-B715E8F0C57D

852866

Figs 2A–D , 3 , 4

##### Etymology.

“*verna*” refers to spring-born.

##### Holotypus.

China, • Jilin Province, Tonghua City, Ji’an City, Yushan Park, 8 May 2023, 41°08'01"N, 126°10'45"E, alt. 280 m, Zheng-Qing Chen, CZQ23050801 (FJAU65117).

##### Diagnosis.

The main characteristic of *Conocybeverna* includes a straight to reflexed edge of the pileus after maturity, with no surface pubescence. The basidiospores exhibit a suprahilar depression and have an oblong, subcylindrical shape with a slightly thin wall. The basidia are 2-spored.

##### Description.

Basidioma mycenoid. Pileus diameter 0.5–2.5 cm, initially paraboloid, nearly hemispherical, margin deflexed, matured obtusely conical, campanulate, margin straight to reflexed. Pileus initially beige (RAL1001) to ivory (RAL1014), matured light ivory (RAL1015), powdery yellow (RAL1034) to ochre brown (RAL8001), surface hygrophanous, pubescence absent, when moist, it exhibits striae, which disappear upon slight drying, margin undulate. Context thin, ivory (RAL1014) to beige (RAL1001), no specific odor or taste. Lamellae adnexed to narrowly adnate, ventricose, crowded, unequal in length, ivory (RAL1014), powdery yellow (RAL1034) to ochre brown (RAL8001), smooth margin. Stipe 2.0–8.0 cm long, 1.0–3.0 mm thick, cylindrical, slightly thicker downward, ivory (RAL1014) to ochre brown (RAL8001), deer brown (RAL8007), surface pruinose and short pubescent, longitudinally fibrous striate, subbulbous at the base.

**Figure 2.**
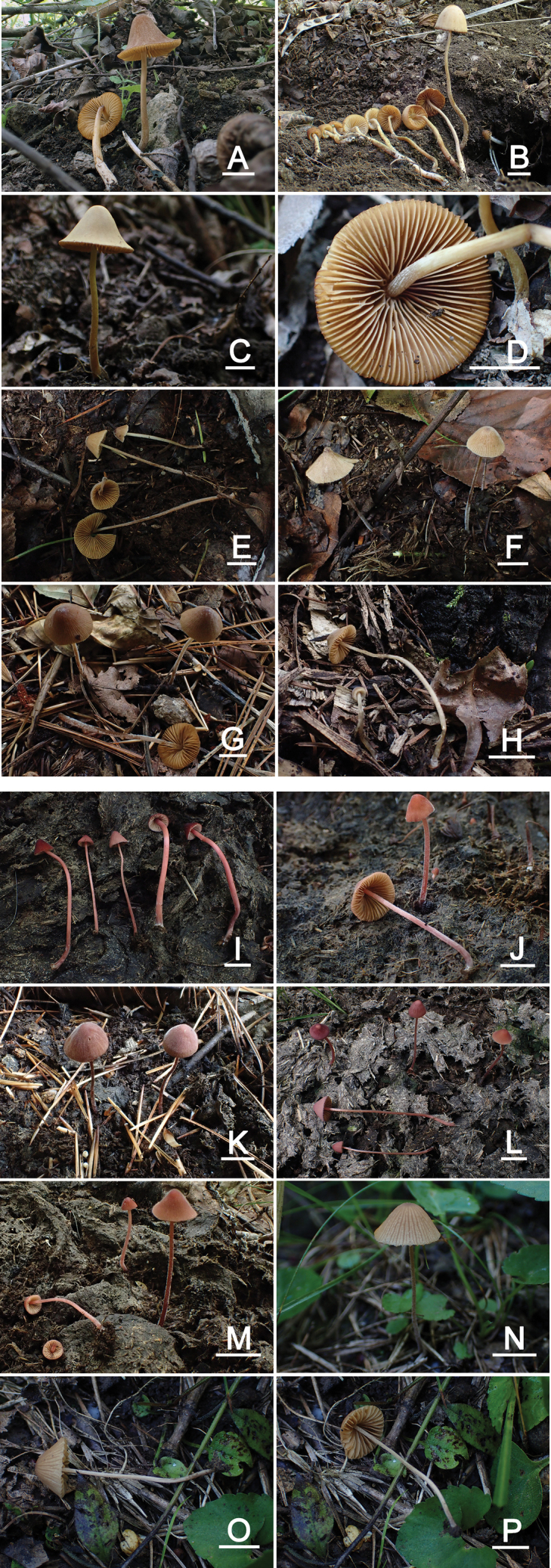
Basidiomata of Conocybesect.Pilosellae species **A***C.verna* (FJAU65117 T) **B***C.verna* (FJAU65118) **C, D***C.verna* (FJAU65119) **E, F***C.angulispora* (FJAU65120 T) **G***C.angulispora* (FJAU65122) **H***C.angulispora* (FJAU65121) **I***C.rubrocyanea* (FJAU65123 T) **J***C.rubrocyanea* (FJAU71654) **K***C.rubrocyanea* (FJAU71658) **L***C.rubrocyanea* (FJAU71652) **M***C.rubrocyanea* (FJAU71650) **N–P***C.hexagonospora* (FJAU71661), Scale bars: 1 cm, T = holotype.

***Basidiospores*** (60/3/3) (10–)11–15.5(–16) × (5.5–)6–8.5(–9) μm, Q=(1.65–)1.71–2.07(–2.21), Qm = 1.86(±0.10), with a suprahilar depression, oblong, subcylindrical, wall slightly thin, containing oil droplets, germ pore diameter 0.5–2.0 μm. Basidiospores in 5% KOH solution appear ochre brown (RAL8001) to copper brown (RAL8004). Basidia (20–)21–33(–35) × (7–)8–11 μm, clavate, 2-spored, sterigmata 3–7 μm long, basidia with vacuolar contents. Cheilocystidia (16–)17–25(–26) × (6–)7–11(–13) μm, lecythiform, with capitula 3–6 μm wide. Caulocystidia ellipsoid to oblong, lageniform, long-necked lageniform, subcylindrical, clavate, narrowly utriform to utriform, fusiform, conical, nettle hair-shaped, (9–)10–50(–53) × 5–12 μm, with capilliform elements reaching up to 80 μm, among which rare lecythiform cystidia are mixed. Pileipellis hymeniform, composed of (23–)31–63(–65) × (14–)15–22(–23) μm sphaeropedunculate elements, with yellow pigments at the base. Pileocystidia absent. All structures have clamp connections. Weakly positive reaction with ammonia forming rhomboid crystals.

**Figure 3.**
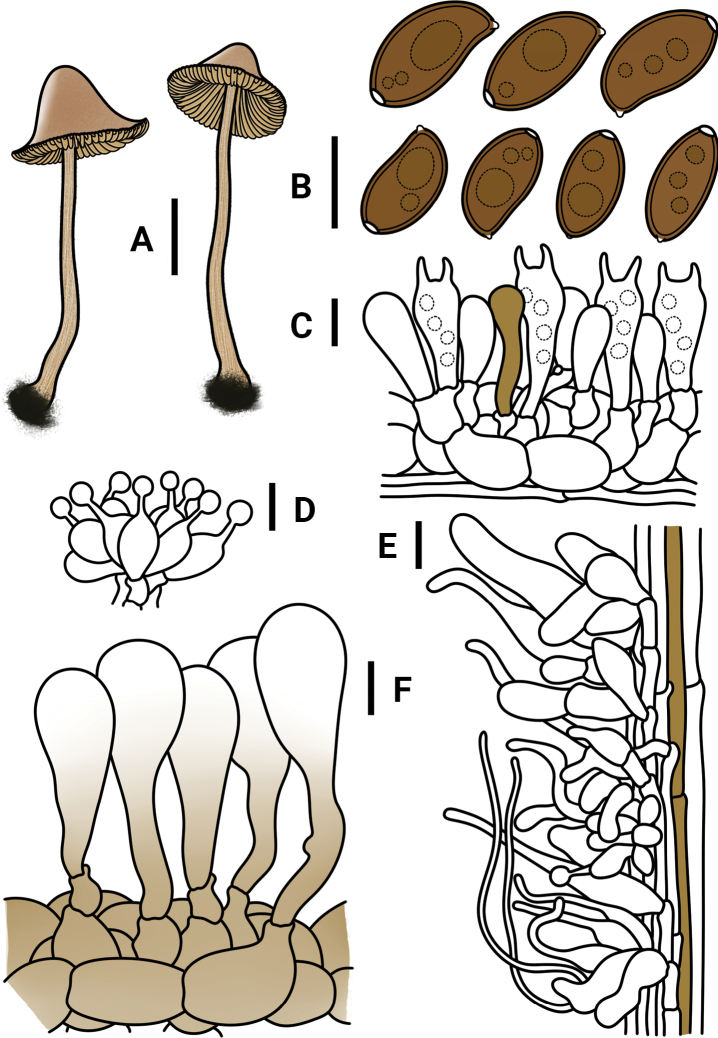
*Conocybeverna* (FJAU65117) **A** basidiomata **B** basidiospores in KOH **C** hymenium and subhymenium **D** cheilocystidia **E** stipitipellis **F** pileipellis. Scale bars: 1 cm (**A**); 10 μm (**B–F**).

##### Habitat.

Found singly or scattered in broad-leaved forests during spring.

##### Known distribution.

Jilin Province, China.

##### Additional specimens measured.

China, • Jilin Province, Tonghua City, Ji’an City, Yushan Park, 8 May 2023, 41°08'01"N, 126°10'45"E, alt. 280 m, Qian-Ru Liu, LQR23050801 (FJAU65118); • Tonghua City, Ji’an City, Jiangkou Village, 9 May 2023, 40°59'37"N, 126°03'02"E, alt. 260 m, Mu Liu, LM230509 (FJAU65119).

**Figure 4.**
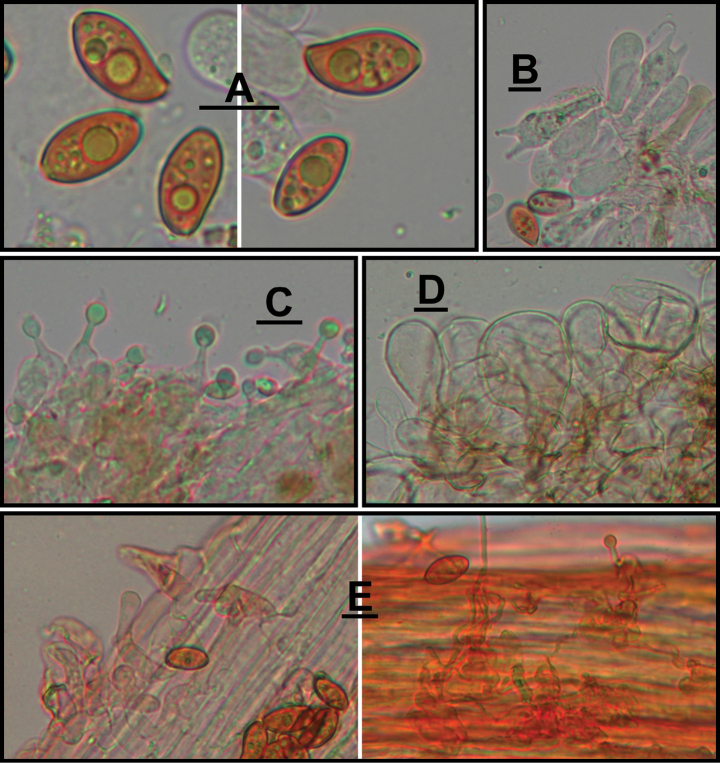
Microscopic structure images of *Conocybeverna* (FJAU65117) **A** basidiospores **B** basidia **C** cheilocystidia **D** pileipellis **E** stipitipellis. Scale bars: 10 μm (**A–E**).

##### Notes.

*Conocybeverna* is classified in sect. Pilosellae primarily due to the presence of non-lecythiform caulocystidia. The distinguishing characteristics of *C.verna* from other 2-spored species in sect. Pilosellae are as follows: *C.verna* differs from *C.bisporigera* (Hausknecht & Krisai) Arnolds in that the latter has a chocolate brown pileus and lentiform basidiospores (Arnolds 2003). The distinction between *C.verna* and *C.caespitosa* (Murrill) Watling is that the latter has basidiospores with a suprahilar plage and basidia measuring 19–24 μm in length, which is shorter than the basidia of *C.verna* (Hausknecht 2009). In contrast to *C.bispora* (Singer) Hauskn., *C.verna* has a pileus without distinct striations, while the basidiospores of *C.bispora* are on average 2 μm shorter (Hausknecht 1998). The distinction between *C.verna* and C.umbellulavar.lednicensis lies in the latter having a striate pileus, and basidia measuring less than 20 μm in length (Hausknecht 2009). Furthermore, *C.verna* is differentiated from *C.leporina* (Velen.) Singer and *C.microrrhiza* Hauskn. by the presence of a pseudorhiza in the latter two, as well as their smaller basidiospores (Singer 1989; Hausknecht 1999). *Conocybeverna* differs from *C.inocybeoides* Watling in that the latter has a pileus with radiating striations and possesses pileocystidia (Watling 1980). Additionally, *C.verna* is distinguished from *C.velutinomarginata* Hauskn. & Zugna and *C.rickenii* (Jul. Schäff.) Kühner by the presence of capilliform pileocystidia in the latter two; *C.velutinomarginata* has a nearly spherical pileus, while *C.rickenii* has a grayish-brown pileus (Kühner 1935; Hausknecht 2009). *Conocybeverna* can be differentiated from *C.siliginea* (Fr.) Kühner by the latter’s lime-colored pileus and lecythiform pileocystidia (Kühner 1935). Finally, the distinction between *C.verna* and *C.gigasperma* Enderle & Hauskn. lies in the latter’s basidiospores measuring 18.3–20.1 μm in length, which are larger than those of *C.verna*, and the presence of pileocystidia (Hausknecht and Enderle 1992). *Conocybeverna* is also distinguished from *C.sinobispora* T. Bau & H.B. Song, as the latter has a striate pileus and cylindrical to lageniform pileocystidia (Song and Bau 2023).

In terms of phylogeny, *C.verna* is closely related to *C.ingridiae* and C.ochrostriatavar.favrei. However, *C.ingridiae* has a pileus with distinct striations and basidiospores measuring 9.6–10.5 μm in length, while C.ochrostriatavar.favrei also has a striate pileus and possesses 4-spored basidia, making them easily distinguishable (Hausknecht 2009). Among these similar species, the following have been sequenced and are clearly separated in the phylogeny: *C.bisporigena*, *C.bispora*, *C.ingridiae*, *C.microrrhiza*, *C.velutinomarginata*, *C.rickenii*, *C.siliginea*, and *C.sinobispora*.

#### ﻿ Conocybe angulispora

Taxon classificationFungiAgaricalesBolbitiaceae

T. Bau & H. B. Song sp. nov.

965CC29D-6A41-5A93-9B8B-9D4DC413344C

852867

Figs 2E–H , 5 , 6

##### Etymology.

“*angulispora*” refers to basidiospores that are angular and submitriform or slightly hexagonal in shape.

##### Holotypus.

China, • Jilin Province, Jilin City, Jiaohe City, Shansongling, 26 August 2023, 43°32'25"N, 127°02'21"E, alt. 550 m, Hong Cheng, C2382612 (FJAU65120).

##### Diagnosis.

*Conocybeangulispora* basidiospores are lentiform, frontal view slightly hexagonal or submitriform, side view ellipsoid to oblong, ovoid, amygdaliform, basidia are 4(2)-spored, and pileocystidia are abundant.

##### Description.

Basidioma mycenoid. Pileus diameter 0.5–2.5 cm, initially paraboloid to obtusely conical, later conical to broadly conical, edge straight, undulate. In early stages, pileus center color ranges from signal brown (RAL8002) to mahogany brown (RAL8016), with slightly lighter color at the edges, brown beige (RAL1011), sandy yellow (RAL1002) to maize yellow (RAL1001). When mature, pileus center color changes to reddish-brown (RAL8012) to mahogany brown (RAL8016), while the edge remains brown beige (RAL1011) and ivory (RAL1014). Pileus hygrophanous, distinctly pubescent, with striations extending to the center. Context thin, ivory (RAL1014) to light ivory (RAL1015), no specific odor or taste. Lamellae adnexed to narrowly adnate, ventricose, slightly crowded, unequal in length, sandy yellow (RAL1002) to ochre brown (RAL8001), with smooth edges. Stipe length 2.5–5.0 cm, thick 1.0–2.0 mm, cylindrical, light ivory (RAL1015), sandy yellow (RAL1002) to signal brown (RAL8002), surface covered with pubescent, longitudinally fibrous striations, subbulbous base.

**Figure 5.**
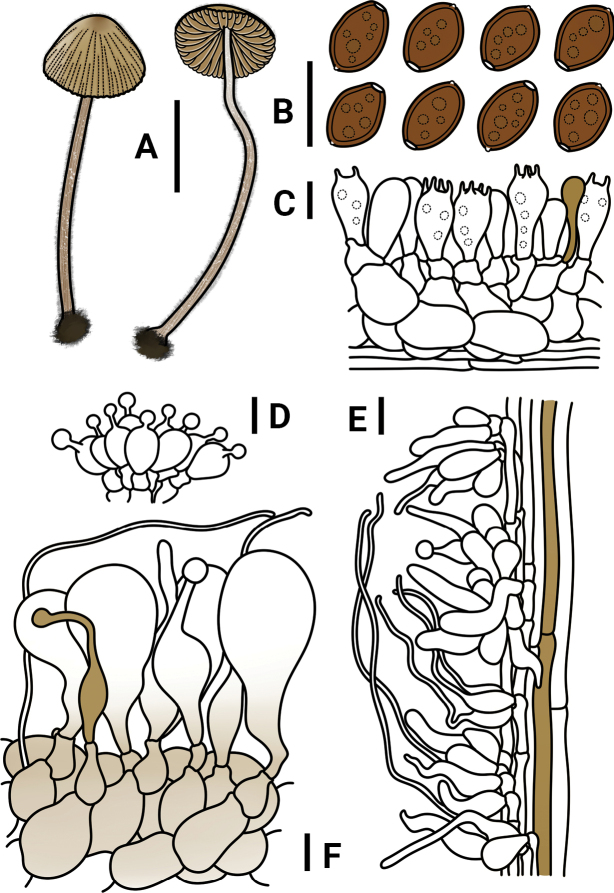
*Conocybeangulispora* (FJAU65120) **A** basidiomata **B** basidiospores in KOH **C** hymenium and subhymenium **D** cheilocystidia **E** stipitipellis **F** pileipellis. Scale bars: 1 cm (**A**); 10 μm (**B–F**).

***Basidiospores*** (60/3/3) 8–10(–10.5) × 5.5–6.5 × (4.5–)5–6 μm, Q=(1.35–)1.39–1.76(–1.83), Qm = 1.57(±0.11), lentiform, angular and submitriform or slightly hexagonal in frontal view, ellipsoid to oblong, ovoid, or amygdaliform in side view, with partially thick walls and containing oil droplets, germ pore diameter 0.5–2.0 μm, basidiospores in 5% KOH solution ochre brown (RAL8001) to copper brown (RAL8004) in KOH. Basidia 14–24(–25) × (8–)9–11(–12) μm, broadly clavate to clavate, 4(2)-spored, sterigmata 2–6 μm long, basidia with vacuolar contents. Cheilocystidia 13–22 × 6–10(–11) μm, lecythiform, with capitula 3–6 μm wide. Caulocystidia ellipsoid to oblong, lageniform, long-necked lageniform, nettle hair-shaped, narrowly conical, fusiform, cylindrical, clavate, narrowly utriform to utriform, (10–)11–42(–45) × (4–)5–9 μm, capilliform cystidia can exceed 100 μm, among which rare lecythiform cystidia are mixed at the apex. Pileipellis hymeniform, composed of (25–)28–62(–66) × 15–34(–36) μm broadly clavate, spheropedunculate, and obpyriform elements, with yellow pigment at the base. Pileocystidia abundant, (22–)23–58(–60) × 5–18(–19) μm, lageniform to long-necked lageniform, lecythiform, tibiiform, and nettle hair-shaped, capilliform cystidia can exceed 100 μm. Clamp connections are rare in all tissues. Shows negative reaction with ammonia solution.

**Figure 6.**
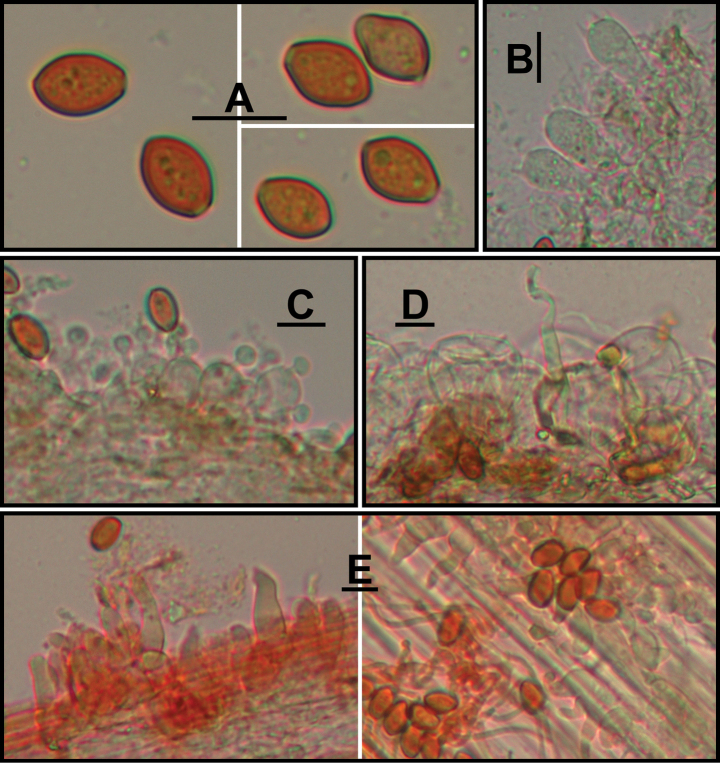
Microscopic structure images of *Conocybeangulispora* (FJAU65120) **A** basidiospores **B** basidia **C** cheilocystidia **D** pileipellis **E** stipitipellis. Scale bars: 10 μm (**A–E**).

##### Habitat.

In summer, they grow scattered or in groups in the humus layer of mixed forests.

##### Known distribution.

Jilin Province, China.

##### Additional specimens measured.

China, • Jilin Province, Jilin City, Jiaohe City, Laoyeling, 28 July 2023, 43°40'57"N, 127°11'58"E, alt. 430 m, Xia Wang, W23072815 (FJAU65121); • Jilin City, Jiaohe City, Shansongling, 26 August 2023, 43°32'09"N, 127°02'23"E, alt. 530 m, Hong Cheng, C2382621 (FJAU65122).

##### Notes.

In some species of section Pilosellae, the frontal view of basidiospores appears slightly hexagonal, which can be easily confused with *C.angulispora*. The difference between *C.angulispora* and *C.hexagonospora* is that *C.hexagonospora* lacks distinct pubescence on the pileus and has rare pileocystidia, making it easy to differentiate (Hausknecht 1993). Additionally, the ITS sequence similarity between *C.angulispora* and *C.hexagonospora* is 91.2%. *Conocybeangulispora* can be distinguished from *C.brunneidisca* by the larger length of basidiospores in *C.brunneidisca*, which can reach 9.9–12.1 μm, and it is found in fertile grasslands or dung (Hausknecht and Contu 2007). *Conocybeangulispora* can be differentiated from *C.pulchra* (Clem.) Hauskn., Krisai & Voglmayr by the length of basidiospores, which measures 11.5–15 μm in *C.pulchra*, and *C.pulchra* lacks pileocystidia (Hausknecht et al. 2004). The difference between *C.angulispora* and *C.lentispora* Singer is that the basidiospores of *C.lentispora* are shorter than 7 μm and broadly ellipsoid in shape (Hausknecht 2005). *Conocybeangulispora* can be differentiated from *C.brunneoaurantiaca* K.A. Thomas, Hauskn. & Manim. such that *C.brunneoaurantiaca* lacks pubescence on the pileus and pileocystidia (Hausknecht 2009; Thomas et al. 2001).

#### ﻿ Conocybe rubrocyanea

Taxon classificationFungiAgaricalesBolbitiaceae

T. Bau & H. B. Song sp. nov.

964E7581-2800-5DF3-8B8E-6CF219508F58

852868

Figs 2I–M , 7 , 8

##### Etymology.

“ *rubrocyanea* “ refers to basidiomata that have a reddish hue when fresh and a bluish hue when dry.

##### Holotypus.

China, • Jilin Province, Jilin City, Jiaohe City, Shansongling, 30 July 2023, 43°32'14"N, 127°01'33"E, alt. 610 m, Shi-En Wang, E2307268 (FJAU65123).

##### Diagnosis.

*Conocyberubrocyanea*, when fresh, displays a mainly red color on the pileus, transitioning to blue upon drying. Basidiospores are lentiform, ellipsoid to oblong, frontal view near hexagonal, side view phaseoliform, cheilocystidia clavate, utriform, ellipsoid, or fusiform on one side near the edge of the pileus, and lecythiform on the side near the stipe, and some pileipellis cells contain blue lilac pigment.

##### Description.

Basidioma mycenoid. Pileus diameter 0.5–2.0 cm, initially hemispherical, conical, later obtusely conical, with straight, undulate margin. When fresh, pileus salmon orange (RAL2012), antique pink (RAL3014) to rose (RAL3017), tomato red (RAL3013) to pearl ruby red (RAL3032), and when dry, it becomes slate gray (RAL7015), brown gray (RAL7013) to cobalt blue (RAL5013). Pileus hygrophanous, covered in distinct pubescence and striations that extend up to one-third towards the center. Context thin, salmon orange (RAL2012) to light ivory (RAL1015), no specific odor or taste. Lamellae ventricose, adnexed to narrowly adnate, moderately crowded, unequally long, initially light ivory (RAL1015) to ivory (RAL1014), later pastel yellow (RAL1034) to ochre-brown (RAL8001), with inconspicuous, slightly eroded edges. Stipe 2.0–8.0 cm long, 1.0–4.0 mm thick, cylindrical, clay brown (RAL8003), rose (RAL3017), antique pink (RAL3014) to pearl ruby red (RAL3032), surface pruinose and pubescent, longitudinally striate, base bulbous.

**Figure 7.**
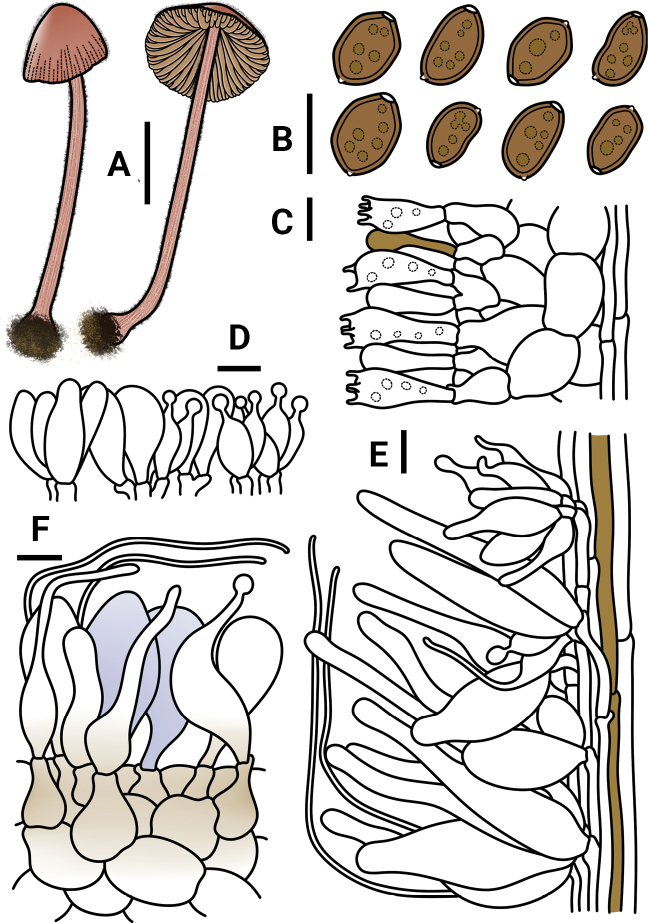
*Conocyberubrocyanea* (FJAU65123) **A** basidiomata **B** basidiospores in KOH **C** hymenium and subhymenium **D** cheilocystidia **E** stipitipellis **F** pileipellis. Scale bars: 1 cm (**A**); 10 μm (**B–F**).

***Basidiospores*** (60/3/3) 8–11.5(–12.5) × 5–7.5 × 5–6(–6.5) μm, Q=(1.33–)1.42–2.08(–2.14), Qm = 1.76(±0.17), lentiform, ellipsoid to oblong, frontal view near hexagonal, side view phaseoliform, slight constriction at center, with thick walls, containing oil droplets, germ pore diameter 0.5–2.0 μm, basidiospores in KOH solution ochre brown (RAL8001) to copper brown (RAL8004). Basidia (13–)15–26(–27) × 8–11(–12) μm, broadly clavate to clavate, 4(2)-spored, with sterigmata measuring 2–6 μm in length, basidia contain vacuolar contents. Cheilocystidia (14–)15–27(–28) × (6–)7–14(–15) μm, clavate, utriform, ellipsoid, or fusiform on one side near the edge of the pileus, and lecythiform on the side near the stipe, with capitula 3–6 μm wide. Caulocystidia elliptical to oblong, lageniform, long-necked lageniform, nettle hair-shaped, conical, fusiform, cylindrical, clavate, narrowly utriform to utriform, (10–)12–82(–85) × (5–)6–16 μm, capilliform cystidia may exceed 100 μm, with rare occurrences of lecythiform and sub-lecythiform cystidia at the apex. Pileipellis hymeniform, composed of spheropedunculate and fusiform cells (25–)27–53(–54) × (14–)15–28(–29) μm, some containing blue lilac (RAL4005) pigment, with yellow pigment at the base. Pileocystidia (21–)23–55(–60) × 4–23 μm, with long-necked lageniform, lecythiform, cylindrical, and nettle hair-shaped forms, and capilliform cystidia can exceed 100 μm. Clamp connections are rare in all tissues. It shows a positive reaction with ammonia, forming diamond-shaped crystals.

**Figure 8.**
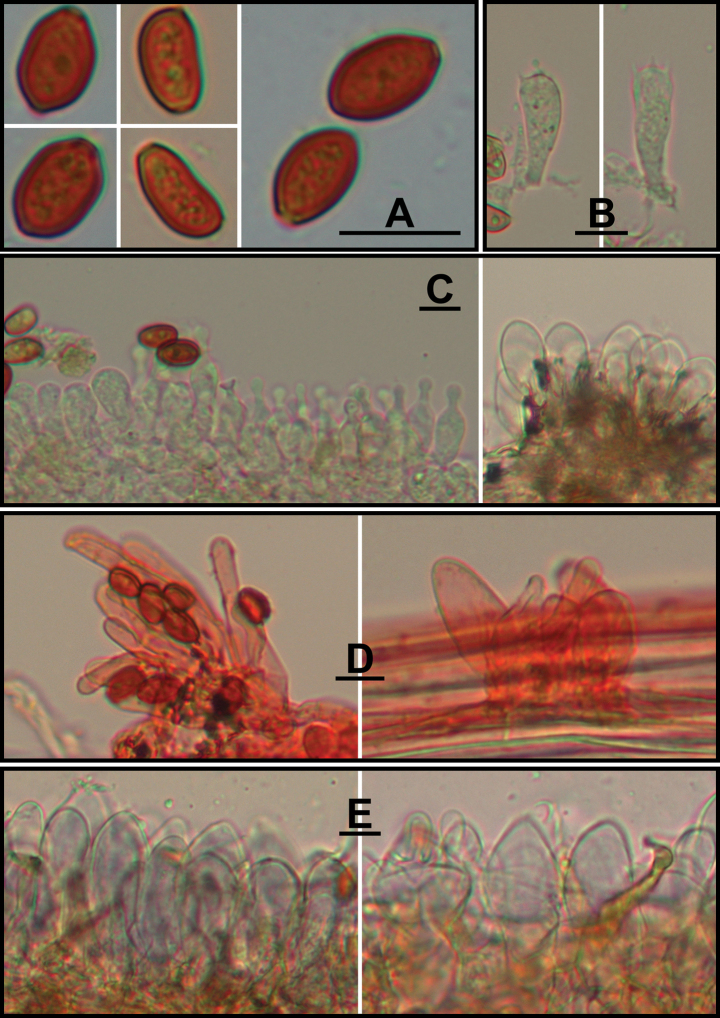
Microscopic structure images of *Conocyberubrocyanea* (FJAU65123) **A** basidiospores, **B** basidia **C** cheilocystidia **D** stipitipellis **E** pileipellis. Scale bars: 10 μm (**A–E**).

##### Habitat.

Scattered or grouped in mixed forests during the summer season, on cow dung.

##### Known distribution.

Jilin Province, China.

##### Additional specimens measured.

China, • Jilin Province, Jilin City, Jiaohe City, Shansongling, 26 July 2022, 43°32'02"N, 127°02'36"E, alt. 580 m, Han-Bing Song, S22072618 (HMJAU64964); • Jilin City, Jiaohe City, Shansongling, 29 July 2023, 43°32'20"N, 127°03'09"E, alt. 530 m, Shi-En Wang, E2307247 (FJAU71648); • Jilin City, Jiaohe City, Shansongling, 30 July 2023, 43°32'20"N, 127°01'50"E, alt. 550 m, Shi-En Wang, Xia Wang, Si-Ying Li, W23073002 (FJAU71649), W23073003 (FJAU71650), W23073004 (FJAU71651), E2307277 (FJAU71652), L23073033 (FJAU71653); • Jilin City, Jiaohe City, Shansongling, 26 August 2023, 43°32'26"N, 127°02'23"E, alt. 550 m, Zheng-Qing Chen, Mu Liu, Hong Cheng, Q2382626 (FJAU71654), LM230864 (FJAU71655), C2382603 (FJAU71656), C2382605 (FJAU71657), C2382611 (FJAU71658), C2382615 (FJAU71659); • Jilin City, Huadian City, Redstone National Forest Park, 28 August 2023, 42°58'08"N, 127°03'36"E, alt. 430 m, Xian-Yan Zhou, Y2382804 (FJAU71660).

##### Notes.

*Conocyberubrocyanea* can be differentiated from species with near hexagonal basidiospores in sect. Pilosellae, such as *C.hexagonospora*, *C.brunneidisca*, *C.lentispora*, *C.brunneoaurantiaca*, *C.pulchra* and *C.angulispora*, by presence of red color tone on the pileus (Hausknecht 2009). *Conocyberubrocyanea* is closely related to *C.incarnata* (Jul. Schäff.) Hauskn. & Arnolds and *C.muscicola*, and they are easily confused in macroscopic morphology. However, *C.incarnata* and *C.muscicola* basidiospores are not lentiform or hexagonal, and pileipellis cells lack blue lilac pigment (Arnolds and Hausknecht 2003).

#### ﻿ Conocybe hexagonospora

Taxon classificationFungiAgaricalesBolbitiaceae

Métrod ex Hauskn. & Enderle

AC2756D9-91EB-59BA-9E90-B08E274BE41D

Figs 2N–P , 9 , 10

##### Description.

Basidioma mycenoid. Pileus diameter 1.0–1.5 cm, obtusely conical, edge straight, undulate, center signal brown (RAL8002) to deer brown (RAL8007), fading towards the edge, brown beige (RAL1011) to ivory (RAL1014), pileus hygrophanous, smooth, striate towards the center. Context thin, ivory (RAL1014) to light ivory (RAL1015), no specific odor or taste. Lamellae adnexed to narrowly adnate, ventricose, slightly loosely, unequal in length, beige (RAL1001) to sandy yellow (RAL1002), with smooth margins. Stipe length 3.5–4.0 cm, width 0.5–1.5 mm, cylindrical, brown beige (RAL1011) to sandy yellow (RAL1002), surface pubescent, longitudinally fibrous striate, subbulbous at the base.

**Figure 9.**
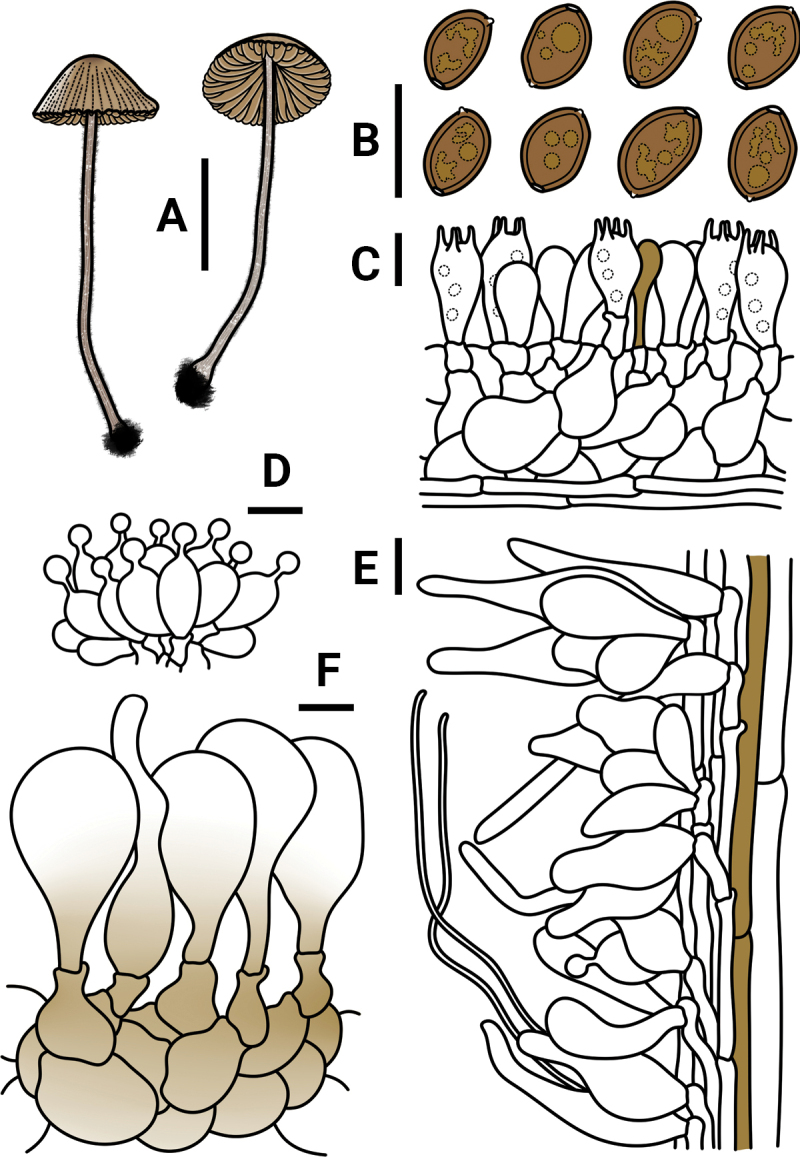
*Conocybehexagonospora* (FJAU71661) **A** basidiomata **B** basidiospores in KOH **C** hymenium and subhymenium **D** cheilocystidia **E** stipitipellis **F** pileipellis. Scale bars: 1 cm (**A**); 10 μm (**B–F**).

***Basidiospores*** (40/1/1) 7.5–9.5(–10) × 5.5–6.5 × 5–6 μm, Q=(1.32–)1.34–1.78(–1.80), Qm = 1.49(±0.11), lentiform, frontal view nearly hexagonal or submitriform, side view ellipsoid to oblong, thick-walled, containing oil droplets, germ pore diameter 0.5–1.5 μm. Basidiospores in 5% KOH solution ochre brown (RAL8001) to copper brown (RAL8004). Basidia (14–)15–21(–22) × 8–10 μm, broadly clavate to clavate, 4-spored, with sterigmata length 3–6 μm, basidia contain vacuolar contents. Cheilocystidia (13–)15–21 × 7–10(–11) μm, lecythiform, with capitula 3–6 μm wide. Caulocystidia are ellipsoid to oblong, lageniform, long-necked lageniform, nettle hair-shaped, narrowly conical, fusiform, cylindrical, clavate, narrowly utriform to utriform, measuring (20–)22–55(–57) × (5–)6–16 μm, capilliform cystidia can reach a length of 100 μm, with rare lecythiform cystidia mixed in. Pileipellis hymeniform, consists of spheropedunculate and obpyriform cells, 29–48(–50) × (18–)19–27(–30) μm, with yellow pigment at the base. Pileocystidia are rare and lageniform in shape. All tissues exhibit clamp connections. It shows a negative reaction to ammonia solution.

**Figure 10.**
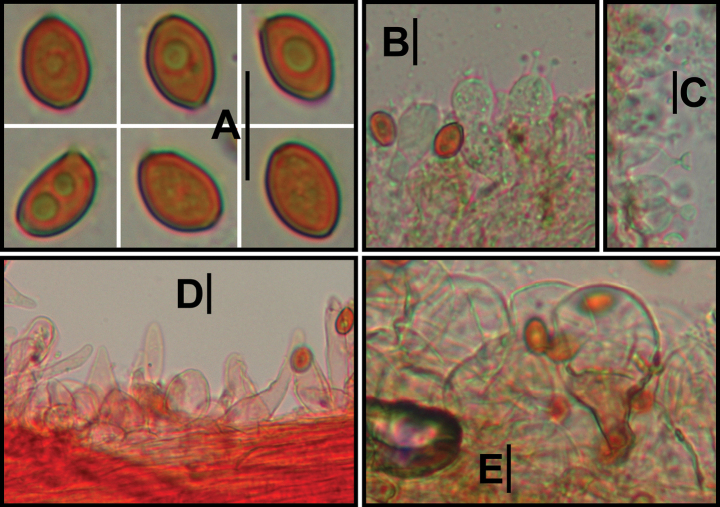
Microscopic structure images of *Conocybehexagonospora* (FJAU71661) **A** basidiospores **B** basidia **C** cheilocystidia **D** stipitipellis **E** pileipellis. Scale bars: 10 μm (**A–E**).

##### Habitat.

Solitary in mixed forests during autumn.

##### Known distribution.

Asia: China, Russia; Europe: Sweden, Finland, Latvia, Hungary, Germany, Austria (Holotype), Belgium, United Kingdom, France, Italy (Hausknecht 2009).

##### Additional specimens measured.

China, • Jilin Province, Siping City, Yitong Manchu Autonomous County, 7 September 2023, 43°35'58"N, 125°12'12"E, alt. 290 m, Han-Bing Song, S23090710 (FJAU71661).

##### Notes.

Although this species does not have gene sequences in the NCBI database, its macroscopic and microscopic structures are consistent with those of *C.hexagonospora*, leading to its identification as *C.hexagonospora*. There are also some species in sect. Pilosellae with basidiospores’ shapes similar to *C.hexagonospora*, but they are distinct species, differentiated as follows: The difference between *C.hexagonospora* and *C.brunneidisca* is that the latter has longer basidiospores, reaching a length of 9.9–12.1 μm, and the pileus color and habitat are also different (Hausknecht and Contu 2007). The difference between *C.hexagonospora* and *C.pulchra* is that the latter has basidiospores measuring 11.5–15 μm in length and lacks pileocystidia (Hausknecht et al. 2004). The difference between *C.hexagonospora* and *C.lentispora* is that the latter has basidiospores with a length smaller than 7 μm and are broadly ellipsoid (Hausknecht 2005). Meanwhile, the difference between *C.hexagonospora* and *C.brunneoaurantiaca* is that *C.brunneoaurantiaca* has cheilocystidia reaching up to 30 μm and lacks pileocystidia (Thomas et al. 2001). In the phylogenetic tree, *C.hexagonospora* and C. *angulispora* are sister taxa to each other, but their ITS sequence similarity is only 91%. *Conocybeangulispora* has distinct pubescence on its pileus, allowing for differentiation from *C.hexagonospora*. Of these similar species, the following are sequenced and clearly separate in the phylogeny: *C.hexagonospora*, *C.brunneidisca*, *C.angulispora*.

### ﻿Key to Chinese Species of ConocybeSect.Pilosellae

**Table:** 

1	2-spored	**2**
–	4-spored	**6**
2	Pileus unstriated or not distinct	**3**
–	Pileus striated	**4**
3	Tibiiform pileocystidia present	***C.siliginea***
–	Tibiiform pileocystidia absent	***C.pseudocrispa***
4	Basidiospores with suprahilar depression	***C.verna***
–	Basidiospores with suprahilar plage	**5**
5	Pileus blackish in color	***C.bisporigera***
–	Pileus yellowish in color	***C.sinobispora***
6	Basidiospores nearly hexagonal	**7**
–	Basidiospores not hexagonal	**10**
7	Pileus reddish in color	***C.rubrocyanea***
–	Pileus lacking a red color	**8**
8	Average length of basidiospores can reach 12 μm	***C.brunneidisca***
–	Average length of basidiospores is less than 10 μm	**9**
9	Pileus pubescence distinct	***C.angulispora***
–	Pileus pubescence absent or indistinct	***C.hexagonospora***
10	Pseudorhiza present	***C.incarnata***
–	Pseudorhiza absent	**11**
11	Germ pore absent or not distinct	***C.pilosella***
–	Germ pore present	**12**
12	Basidiospores phaseoliform, reniform	***C.reniformis***
–	Basidiospores never phaseoliform	**13**
13	Pileus unstriated	**14**
–	Pileus striated	**15**
14	Waxy crystals precipitate upon drying	***C.ceracea***
–	No crystallization occurs upon drying	***C.fuscimarginata***
15	Pileus densely pubescent	**16**
–	Pileus pubescence absent or slight	**17**
16	Pileus salmon orange when young	***C.muscicola***
–	Pileus blackish-red when young	***C.pilosa***
17	Lamellae edge serrate	***C.hydrophila***
–	Lamellae edge not serrate	**18**
18	Basidiospores lentiform	**19**
–	Basidiospores never lentiform	**20**
19	Length of basidiospores may exceed 15 μm, 11–16 μm	***C.nitrophila***
–	Length of basidiospores may be less than 10 μm, 9–13 μm	***C.velutipes***
20	Grows on cow dung	***C.rufostipis***
–	Grows in meadows	**21**
21	Pileus honey yellow	***C.siennophylla***
–	Pileus brown beige	***C.moseri***

## ﻿Discussion

Building on the phylogenetic framework of Tóth et al. (2013) and Song and Bau (2023), a phylogenetic tree was reconstructed for sect. Pilosellae incorporating materials from Jilin province and using ITS, nrLSU, and *tef1-α*. The analysis revealed the presence of three new species and a new record for China. The new species are *C.verna*, *C.angulispora*, and *C.rubrocyanea*, while the newly recorded species is *C.hexagonospora*. *Conocybeverna* is found in spring in broad-leaved forests and has a campanulate pileus, which lacks pubescence. It has 2-spored basidia and basidiospores are with a suprahilar depression, which distinguishes it from other species in sect. Pilosellae. On the other hand, *C.angulispora* is found in mixed forests and has an obtusely conical pileus with distinct pubescence, and basidiospores are lentiform, angular, submitriform, or slightly hexagonal in frontal view. *Conocyberubrocyanea* grows on cow dung, with macroscopic features similar to *C.incarnata*, but the basidiospores of *C.rubrocyanea* are lentiform in shape, and the pileipellis contains blue lilac pigment. Both dried specimens in water and KOH solution secrete blue-purple pigments. Among the three new species, *C.rubrocyanea* is a particularly unique species. Its caulocystidia predominantly exhibit a non-lecythiform shape, which aligns with the stipe type in sect. Pilosellae. However, its pileipellis is composed of spheropedunculate and fusiform elements, some of which are partly rostellate, characteristics that are consistent with the classification features of sect. Obscurae Hauskn. & Krisai (Hausknecht and Krisai-Greilhuber 2006). Currently, the sect. Obscurae includes only one species, *C.obscura* Watling, which is found in the Democratic Republic of the Congo in Africa. The caulocystidia of *C.obscura* also conform to the stipe type in sect. Pilosellae, yet it lacks molecular sequences, leaving its taxonomic position unclear. It is uncertain whether *C.obscura* clusters with *C.rubrocyanea*, especially since its basidiospores are neither lentiform nor hexagonal, making them easy to distinguish from those of *C.rubrocyanea* (Watling 1973). Although the morphological features of *C.rubrocyanea* are consistent with other species of sect. Obscurae, its position on the basis of the molecular phylogeny is actually within sect. Pilosellae. If *C.obscura* and *C.rubrocyanea* do not cluster together, this would suggest that pileipellis characteristics are not used as criteria for distinguishing sections. In this case, *C.obscura* would also belong to sect. Pilosellae. To resolve this issue, further research on the holotype specimen of *C.obscura* is necessary before reconsidering the classification of *C.obscura* and *C.rubrocyanea*. In this study the holotype of *C.hexagonospora* lacks a sequence, the macroscopic and microscopic structures of specimen FJAU71661 are consistent with that of *C.hexagonospora*, thus confirming FJAU71661 as *C.hexagonospora* (Hausknecht 2009). Additionally, *C.siliginea* (FJAU71664), collected from Henan Province, grows in greenhouse soil, with a lime-colored pileus and 2-spored basidia. We identified specimen FJAU71662 as *C.velutipes* based on its macroscopic and microscopic structures. Subsequently, we obtained the ITS, nrLSU, and *tef1-α* sequences of specimen FJAU71662, which are similar to those of *C.velutipes* (SZMC-NL-2187), with an ITS similarity of 99.7%, nrLSU similarity of 99.9%, and *tef1-α* similarity of 99.2%. This further supports the correctness of our traditional taxonomic identification of the species. Hausknecht and Contu (2007) has already stated that *C.lenticulospora* is a synonym of *C.brunneidisca*, but since 2007, some have continued to use *C.lenticulospora* as a species name, and it is still treated as an independent species in MycoBank and Index Fungorum. Liu (2018) described and illustrated specimen HMJAU45069 as *C.lenticulospora* Watling, with its ITS sequence showing a similarity of 99.7% to *C.lenticulospora* (SZMC-NL-0923). Therefore, we reexamined HMJAU45069, and its basidiospores measured 9.5–13.5 × 6.5–8.5 × 5.5–7.5 μm, lentiform in shape, with a nearly hexagonal or submitriform frontal view and an ellipsoid to oblong side view. The microscopic features were consistent with those of *C.brunneidisca*, supporting Hausknecht and Contu’s (2007) viewpoint that *C.lenticulospora* is a synonym of *C.brunneidisca*.

Based on morphological classification, Hausknecht and Krisai-Greilhuber (2006) divided sect. Pilosellae into 2 subsections and 11 series, contributing significantly to this field. However, when molecular techniques and phylogenetic methods were applied to taxonomy, the correlation between morphological classification and phylogeny revealed some discrepancies. For instance, *C.cylindracea*, classified under ser. Cylindracea, clustered with *C.brunneidisca*, which is the type species of ser. Lenticulospora. Given that *C.cylindracea* has lentiform and slightly angular basidiospores, we propose placing it in ser. Lenticulospora, a change supported by phylogenetic analysis (Fig. 1). This finding suggests that pileus shape is not a reliable feature for series classification. Similarly, *C.angulispora* and *C.hexagonospora*, discovered in Jilin province, fit the definition of ser. Lenticulospora. Consequently, the branch consisting of *C.brunneidisca*, *C.cylindracea*, *C.angulispora*, and *C.hexagonospora* is now designated as ser. Lenticulospora, based on consistency between morphological and phylogenetic analyses. However, *C.rubrocyanea*, which possesses lentiform basidiospores and a hexagonal frontal view does not cluster with ser. Lenticulospora. This disparity between morphological and phylogenetic congruence is also observed in other series. For example, *C.incarnata*, belonging to ser. Microrrhiza due to its pseudorhiza, clusters with *C.muscicola* and *C.rubrocyanea*, making it challenging to differentiate it from other series. Although all these species share the common feature of a reddish pileus, we do not introduce it as a new series. This decision is based on the extensive description and recording of nearly 60 species within sect. Pilosellae by Hausknecht (2009), with many species lacking sequences. Consequently, it remains uncertain whether other species can cluster with the branch containing *C.rubrocyanea*.

This article primarily introduces three new species from Jilin province and a new record for China. Additionally, a key to differentiate the 22 species within sect. Pilosellae in China is provided. However, the phylogenetic positions of the series within sect. Pilosellae are still uncertain. To address this issue, a substantial number of specimens and sequences are required to identify stable shared characteristics for distinguishing different branches. Further in-depth research is needed to investigate this matter.

## Supplementary Material

XML Treatment for Conocybe verna

XML Treatment for Conocybe angulispora

XML Treatment for Conocybe rubrocyanea

XML Treatment for Conocybe hexagonospora

## ﻿Additional information

### Author ORCIDs

Han-bing Song https://orcid.org/0000-0002-7440-5444

Tolgor Bau https://orcid.org/0000-0003-2461-9345

### Data availability

All the sequences have been deposited in GenBank (https://www.ncbi.nlm.nih.gov) and Mycobank (https://www.mycobank.org). The data presented in this study are deposited in the Zenodo repository, accession number https://doi.org/10.5281/zenodo.14836573.
